# Effects of Air Entrainment on Bacterial Viability in Cement Paste

**DOI:** 10.3390/ma15062163

**Published:** 2022-03-15

**Authors:** Hayeon Kim, H. M. Son

**Affiliations:** Department of Civil and Environmental Engineering, Korea Advanced Institute of Science and Technology, 291 Daehak-ro, Yuseong-gu, Daejeon 34141, Korea; gkdus305@kaist.ac.kr

**Keywords:** AE agent, bacteria, compressive strength, CaCO_3_, Portland cement

## Abstract

This study investigated the effect of air entrainment (AE) on bacterial viability in cementitious materials. Specimens were fabricated with Portland cement, urea, calcium lactate, and ureolytic bacteria, and with varying amounts of an AE agent. Specimens with different amounts of the AE agent were fabricated, and then a compressive strength test, quantitative polymerase chain reaction, X-ray diffraction, and thermogravimetry were used to investigate the mechanical properties, viability of bacteria, and hydrates of the specimens. The highest compressive strength was achieved by the specimen with 0.3% AE agent, while the compressive strength of the specimens decreased considerably when the incorporated AE agent was over 0.6%, due to increased porosity. The quantitative polymerase chain reaction result showed that the cell number of the viable bacteria was increased by incorporation of the AE agent, which also corresponded with an increase in CaCO_3_ due to microbial mineral formation. The obtained result confirmed the positive effect of AE agent incorporation in cementitious materials containing bacterial admixtures, as the viability of bacteria, which play a vital role in self-healing efficiency of concrete, was increased by the space provided by the AE agent in the cement matrix. In addition, the quantity of CaCO_3_ and the compressive strength were highest when 0.3% AE agent was incorporated.

## 1. Introduction

Micro-cracks in concrete are generated by external force, freezing and thawing due to temperature and humidity changes, and drying shrinkage [1,2]. The micro-cracks generated by these factors not only can undermine the structure, but also accelerate the durability reduction of the concrete structure due to an increase in the penetrated hazardous chemical materials [3,4]. Numerous methods have been established to deal with these problems. Coatings using epoxy, asphalt, and other polymeric materials have been used to increase the durability of concrete [5,6,7]. Micro-cracks have also been repaired using a polymer grouting method [5,6,7]. However, such crack-control methods have not been very successful; they not only incompletely heal the crack, but also have an adverse effect on the environment in the case of using polymers. In addition, economic loss may occur due to the additional surface treatment and utilization of the material. Therefore, an efficient method to heal micro-cracks is needed.

Various studies have been conducted to explore viable means of healing cracks via self-healing and tackling the disadvantages of the aforementioned conventional micro-crack-control and repair strategies. In particular, self-healing concrete has recently received attention for its ability to heal sustainably micro-cracks. In particular, self-healing concrete incorporating bacteria has been a topic of many studies [5,6,7,8,9,10,11], due to its excellent crack-healing property [12,13,14,15]. However, the technical constraint which remains unresolved with regard to self-healing concrete is the fact that the cell number of viable bacteria is drastically reduced in the concrete matrix due to internal pore filling in concrete when the concrete is hardening [16]. As an alternative approach, encapsulation of bacteria in a polymeric capsule has been studied to protect bacteria from the pressure caused by concrete hardening [17]. Wang et al., reported that the water permeability of self-healing concrete was significantly reduced by incorporating microcapsules containing bacterial spores [17]. This study examined the performance of concrete containing silica-gel- or polyurethane-encapsulated bacteria, and it was confirmed that the bacteria immobilized with polyurethane had the highest crack healing effect [18]. In addition, a hydrogel was used to encapsulate spores of bacteria, and it was found that cracks greater than 0.5 mm could be healed within 7 days [19].

However, since capsule-type self-healing of concrete requires additional incorporation of materials such as melamine and processes such as polymerization and drying spores for capsule production, it is difficult to produce large quantities of the capsules to be incorporated into concrete. In efforts to address these problems, a method of incorporating the microbial culture medium directly into self-healing concrete has been attracting widespread interest [20,21,22,23]. These studies investigated the mechanical properties of specimens by directly incorporating a bacterial culture medium inoculated with bacteria into concrete as mixing water. It has been reported that when the culture medium was mixed directly with cementitious materials, the compressive strength increased due to internal void-filling by CaCO_3_ produced from microbiologically induced calcium carbonate precipitation (MICP) [20,21,22]. This is closely related to the fact that bacteria survive in the harsh environment in concrete, and the significantly high urea degradation metabolism by bacteria can take place when the culture medium is mixed directly.

Gosh et al. [21] fabricated mortars using a culture medium where *Shewanella* was inoculated [21]. As a result, it was confirmed that incorporating *Shewanella* improved the compressive strength of the mortar by reducing the pore size of the mortar [21]. Bundur et al., also investigated whether the hydration kinetics and compressive strength of mortar changed when the *Sporosarcina. pasteurii* were mixed with a cementitious material, and it was determined that bacteria could survive in a cement matrix without additional processing such as using capsules [23]. Hence, it can be assumed that when the bacterial culture was incorporated directly, the compressive strength was enhanced by the space-filling effect of CaCO_3_ produced by the bacterial metabolism.

However, as reported earlier, direct incorporation of spores reduced the viability of bacteria. As a result, additional studies on enhancing the viability of bacteria and the ability to precipitate CaCO_3_ should be carried out in parallel [16]. Recently, there have been numerous studies on the effect of an AE agent on the properties of concrete [24,25]. In the present study, the effect of incorporation of an AE agent into Portland cement paste and mortar specimens on the bacterial viability and concrete mechanical properties was investigated. Firstly, the zeta potential and pH measurements of the culture media containing varying amounts of the AE agent were determined to investigate the effect of incorporation of the AE agent on the viability of the bacteria in culture media. In addition, specimens with different amounts of the AE agent were fabricated, and then a compressive strength test, quantitative PCR (q-PCR), X-ray diffraction (XRD), and thermogravimetry (TG) were used to investigate the viability of the bacteria and the mechanical and hydration properties of the specimens.

## 2. Experimental Procedure

### 2.1. Materials and Specimen Preparation

When an air-entraining agent (AE agent) is incorporated into the cementitious materials, it improves the dispersion of air bubbles [26]. In addition, the evenly distributed air bubbles can provide a space for microorganisms to live in the extreme environment of cementitious materials. However, the AE agent causes interaction between the air bubbles and cement particles, thereby affecting the changes in the mechanical properties [26]. Hence, the amount of AE agent incorporated in cementitious materials can be an important factor affecting the changes in the physical properties and bacterial viability. In this study, the bacterial growth rate and properties of cementitious materials were measured using the amount of AE agent as a variable. As a bio material, *Sporosarcina pasteurii* (ATCC 11859, Korea Collection for Type Cultures, Jeongeup, South Korea), which is capable of decomposing urea and forming CaCO_3_ by absorbing calcium ions, was used [27]. *S. pasteurii* was inoculated in a tryptic soy broth (TSB)–urea medium (Becton Dickinson, Franklin Lakes, NJ, USA) and was cultured at 30 °C and 200 rpm (revolution per minute) for 24 h. In addition, different ratios of an anion surfactant AE agent (ASCO AE-700, AK Chemtech Co. Ltd., Seoul, South Korea) were used. The chemical composition of the cement, and the mix proportion are summarized in Table 1 and Table 2, respectively. OPC (SUNGSHIN CEMENT CO. LTD, Seoul, South Korea) and sand were used as a binder and fine aggregate, respectively, and medium as a bacterial paste solution was added instead of pure water. In addition, urea (Sigma-Aldrich, St. Louis, MO, USA) and calcium lactate (Sigma-Aldrich, St. Louis, MO, USA) were added for supplying the nutrient, which was required for bacterial CaCO_3_ precipitation metabolism. Mortar specimens were fabricated with a weight ratio of medium:cement:fine aggregate of 0.4:1:1. Specimens with different AE agent contents were prepared (0.0, 0.3, 0.6, and 0.9% AE agent, denoted as A0, A3, A6, and A9, respectively). The procedure employed to fabricate the specimens was as follows. Dry cement and sand were stirred for 5 min to ensure homogeneity. Culture media with different ratios of the AE agent were added to the mixture and mixed for five minutes. The prepared mixture was poured into a mold of 50 × 50 × 50 mm^3^. The prepared mortar and paste specimens were cured in a curing room fixed at 20 °C with a plastic wrap to prevent the evaporation of water. Since culture medium was used as mixing water, the specimen was air-cured without exposed to culture medium.

### 2.2. Test Methods

The zeta potential represents the repulsion between the particles and can be used to measure the dispersibility of particles in a colloid [28]. The zeta potential of the culture media containing inoculated bacteria, in which different amounts of the AE agent had been incorporated was measured using a Nano ZSP/ZEN5602 (Malvern Instruments, Malvern, United Kingdom) to investigate the effect of the AE agent on the bacterial ureolytic ability. The pH of the culture media was measured using a pH measuring instrument (Mettler Tolredo, Columbus, OH, USA). Compressive strengths of specimens with different amounts of AE agent were measured using a universal testing machine according to ASTM C 109. The compressive strengths of the specimens were measured on the 7th, 14th, and 28th days. XRD analysis was performed using a Rigaku D/MAX-2500 (Tokyo, Japan) with Cu-ka, and the scan range was 5–65° at a scan speed 0.5°/min. TG analysis was performed using TGA/DSL/1600LF (Mettler-Toledo, Columbus, OH, USA) in an N_2_ gas environment heated to 10 K/min.

To observe the viability of *S. pasteurii* during hydration, cell extractions from the surface of the paste specimens water-cured for 7 and 14 days, were carried out. The paste specimens (20 g) were powdered and sonicated by a bath-type ultrasonicator (Cole-Parmer, Mumbai, India) (40 KHz; 200 W) with deionized water at a mass ratio of 1:1 to separate the *S. pasteurii* present on the surface or inside the specimens for 5 min. Cell pellets were obtained by centrifuging at 8000× *g* for 10 min, and the DNA extraction process was performed according to the protocol provided by the manufacturer of DNeasy blood and tissue kit (Qiagen, Hilden, German). Quantification of ureA, ureB, and ureC genes that express urease of *S. pasteurii* can be used to estimate the viable cell number during cement hydration. The new forward and reverse primers to target ureA, ureB, and ureC genes were designed using Primer 3 software (Table 3). q-PCR assay was performed with a QuantStudio 3 real-time PCR instrument (Thermo Fisher Scientific, Waltham, MA, USA) based on the SYBR^®^ green detection chemistry method. For the reaction, a Power SYBR green PCR master mix (Applied Biosystems, Waltham, MA, USA), forward/reverse primers, distilled water, and DNA specimens were used. A standard curve was used to determine the cell number, and was obtained with a serial dilution series of plasmid in which the target products were inserted into vectors using TA cloning. q-PCR analysis of all DNA specimens was performed in triplicate to obtain reliable values.

## 3. Results

### 3.1. Effect of Incorporation of an Air Entrainment (AE) Agent on Bacterial Ureolysis in Cultured Media

*S. pasteurii* is a typical Gram-positive bacterium with a negatively charged cell surface, which not only decomposes urea, but also adsorbs calcium, which is a cation [27]. Therefore, the effect of the AE agent on the bacterial metabolism can be determined by comparing the change levels of the zeta potential and the pH of culture medium when the AE agent is mixed into the culture medium.

**Figure 1:**
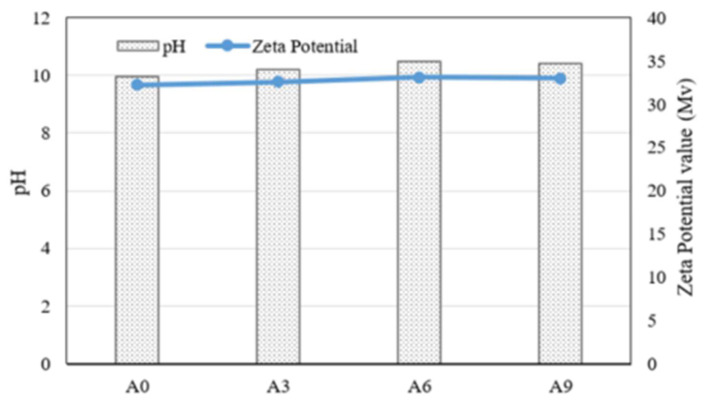
pH and zeta potential of culture media that incorporated different amount of the AE agent at 1 day of incubation.

Figure 1 shows the changes in the pH and zeta potential of medium with the different amounts of the AE agent, respectively. Initial pH of pure medium was 7.0, and A0 (i.e., AE agent was not incorporated) showed a slight alkaline, a pH 9.6. These results indicate that the pH value of medium in which *S. pasteurii* was inoculated was similar to that of the previous study, which showed an alkaline pH of about 9.5 after 1 day of inoculation in the TSB–urea media [27]. This may have been due to the increase in the concentration of OH^−^ and NH_4_^+^ formed by the decomposition of urea, which resulted in an increase in the pH, despite an increase in the amount of CO_2_ formed by the microbial respiration of *S. pasteurii* [27]. On the other hand, when 0.3, 0.6, and 0.9% of AE agent were added, the pH value of culture medium was 9.78, 9.93, and 9.90, respectively. It can be inferred that incorporating the AE agent did not negatively affect the ureolysis metabolism of *S. pasteurii*.

Meanwhile, this change in the pH was closely related to the change in the zeta potential. Indeed, when the pH of a solution is increased by formation of the alkali component, the zeta potential value of the solution decreases due to mobility of the isoelectric point [29]. In addition, since the zeta potential value can help understand the behavior of particle surfaces, the interaction between bacterial cell surfaces and AE agent particles can be inferred by measuring the zeta potential of a bacterial culture medium containing an AE agent. In addition, as the absolute value of the zeta potential increases, it can be said that there is a strong interaction between particles [26]. In particular, from the viewpoint of bacterial growth, the highest absolute value of zeta potential is measured in the exponential phase of bacterial growth [30]. The zeta potential value of A0 was −33.2 Mv. The zeta potentials of A3, A6, and A9 were −34, −35 and −34.7 Mv, respectively (Figure 1). Considering that the zeta potential is closely related to the growth of bacteria cells as the cell walls are negatively charged [31], a similar zeta potential value of the media with bacteria indicated that ureolysis of bacteria was not influenced by incorporation of the AE agent [31]. Nevertheless, as the amount of AE agent was increased, a slight increase in the zeta potential value was observed. A possible explanation lies in the fact that the interaction between bacterial cell surfaces and AE agent particles was relatively changed due to the change in anion properties caused by an increase in the amount of AE agent.

### 3.2. Effect of Incorporation of an AE Agent on the Bacterial Viability and Hydration in Paste Specimens

**Figure 2:**
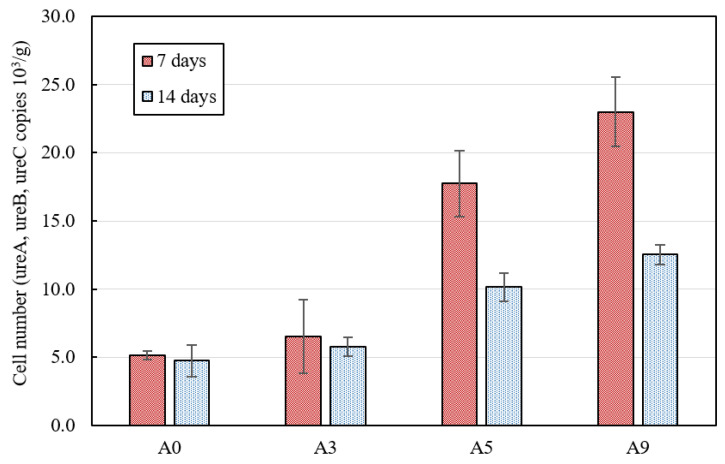
Viability of *S. pasteurii* in the paste specimens on the 7th and 14th days.

The bacterial growth rate can be rapidly decreased during the initial cement hydration and hardening period (from 7th day to 14th day of curing), due to the extreme environment (i.e., high temperature and pressure, hydrate products, etc.) formed by cement hydration [32]. In order to investigate the effect of the AE agent on the bacterial viability during the initial hydration process, the number of viable bacteria in the cementitious materials was measured after the 7th and 14th day of curing. The number of viable *S. pasteurii* cells present in the paste specimens was quantitatively analyzed using q-PCR. q-PCR analysis in this study monitored the amplification of the urea A, B, and C gene regions in real time, by means of newly designed primers used to target only the urease expression gene of *S. pasteurii*. Figure 2 presents the q-PCR results. The results show that the cell number (ureA, ureB, and urea C copies/g, urea, ureB, and ureC (three subunits of urease)) in the paste specimens was found to increase with increasing amount of the AE agent. This shows that as the incorporated AE agent content was increased, the number of bacterial cells capable of producing CaCO_3_ increased. It can be assumed that the incorporation of the AE agent generated pores that provided the space for the bacteria to survive in the cement matrix, and thus destruction of the cell membrane of the bacteria was inhibited by the hydrate in the cement matrix [16]. Although the amount of viable *S. pasteurii* increased as the amount of incorporated AE agent increased, it was found that the cell number of viable *S. pasteurii* decreased on the 14th day compared with that on the 7th day. This is similar to previous results that the viability and CaCO_3_ precipitation ability of bacteria decreased due to a decreased amount of internal voids due to the paste-hardening and densifying processes [16].

**Figure 3:**
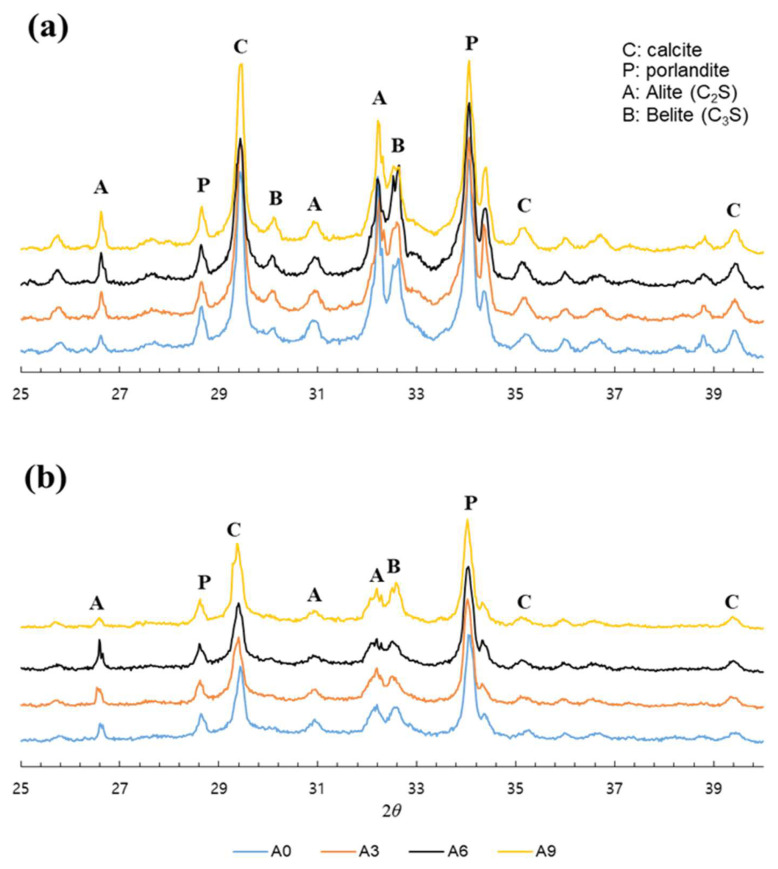
XRD patterns of the paste specimens on the (**a**) 7th and (**b**) 14th days.

**Figure 4:**
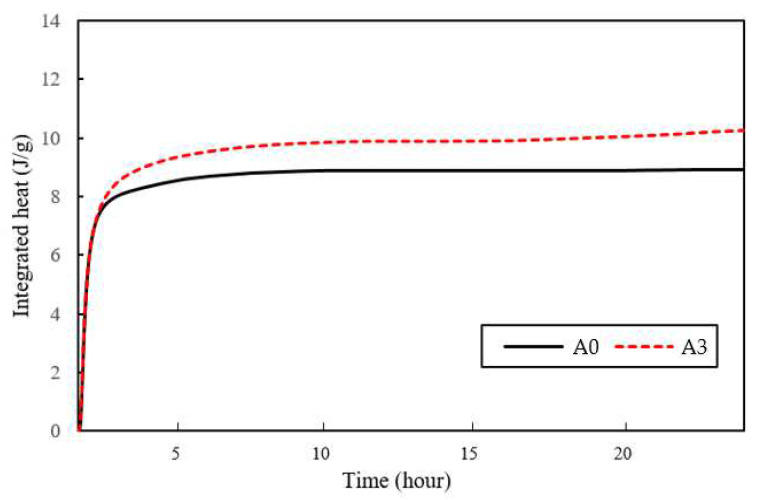
Integral heat outputs applied to the A0 and A3 specimens.

Figure 3 shows the XRD results of the paste specimens on the 7th and 14th days. The effect of the amounts of AE agent on the change in hydrate types was determined by analyzing the XRD results of the specimens A0, A3, A6, and A9 after the 7th and 14th days of curing. There was not significant difference on the hydrate types according to the amount of AE agent. It can be suggested that the amount of AE agent and the incorporation of bacterial culture solution do not significantly affect the change in the type of hydrates. The XRD results show that the CaCO_3_ formed by biomineralization of *S. pasteurii* was calcite. The calcite peaks observed for all specimens show that incorporating the AE agent in the paste specimens did not decrease the amount of calcite formation. Figure 4 shows the integral heat outputs of A0 and A3. The results of the integral heat output were significantly different from the case of not incorporating the AE agent and the case of incorporating 0.3% AE agent. The integral heat of A0 and A3 was 7.91 Joule/g and 12.27 Joule/g, respectively. It can be said that the incorporation of 0.3% AE agent can accelerate the cement hydration. In fact, the bacteria and nutrients contained in the medium are essential for the bacterial CaCO_3_ precipitation metabolism, but it is known that these components can enclose the cement particles and interrupt the contact between cement clinker and water, thereby retarding the initial cement hydration [23,33]. However, the AE agent has a structure with both anion and hydrophobicity, and it attaches to cement particles and relatively stabilizes the air bubbles [26]. Therefore, the incorporation of the AE agent can be expected to accelerate initial hydration by enclosing the cement clinker particles and delaying the contact between cement clinker and nutrients.

**Figure 5:**
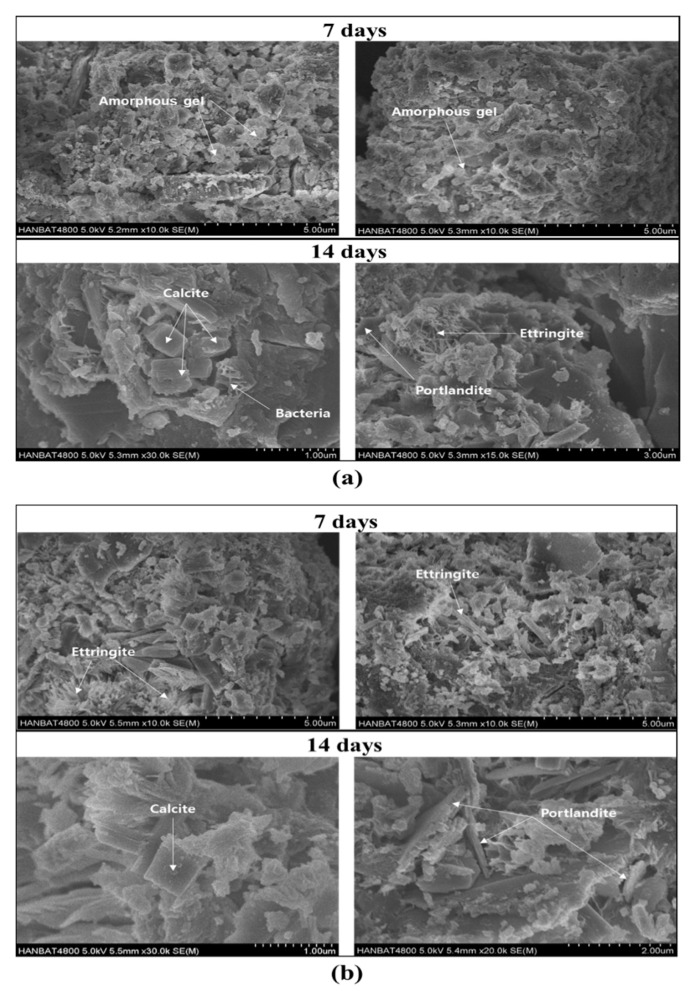
SEM images of the A0 (**a**) and A3 (**b**) paste specimens on the 7th and 14th days.

Furthermore, the results of this integral heat showed a high correlation with the expression of compressive strengths in the specimens. As a result, the incorporation of the AE agent accelerated the hydration of mortar containing *S. pasteurii*, resulting in a higher integral heat output, which consequently increased the compressive strengths. Figure 5 shows SEM images of A0 and A3 specimens on the 7th and 14th day. Both A0 and A3 showed the formation of ettringite, hexagonal calcite, and portlandite-like plates. As shown by the XRD results, only calcite formed by microbial-induced calcite precipitation was found in the CaCO_3_ crystal phase, and vaterite and aragonite were not found. In addition, bacterial cells with a size of 1–10 µm were observed around the minerals. In general, the negatively charged bacterial cell surface can adsorb the metal ion and serve as a nucleation site for mineral precipitation [34]. Through the presence of bacterial cells observed in SEM images, it can be inferred that the addition of an AE agent has a positive effect on the improvement of the bacterial viability and metabolic performance even in the extreme environment of cementitious materials.

### 3.3. Effect of Incorporation of an AE Agent on the Biomineralization of Bacteria in Paste Specimens

**Figure 6:**
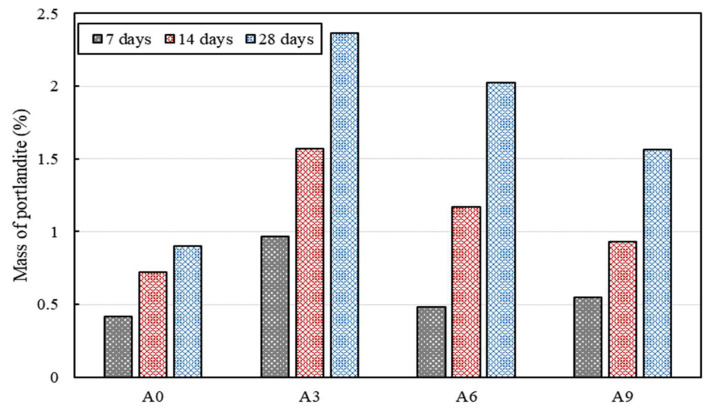
Mass of portlandite in the paste specimens on the 7th, 14^th^, and 28th days, quantified from TG results.

**Figure 7:**
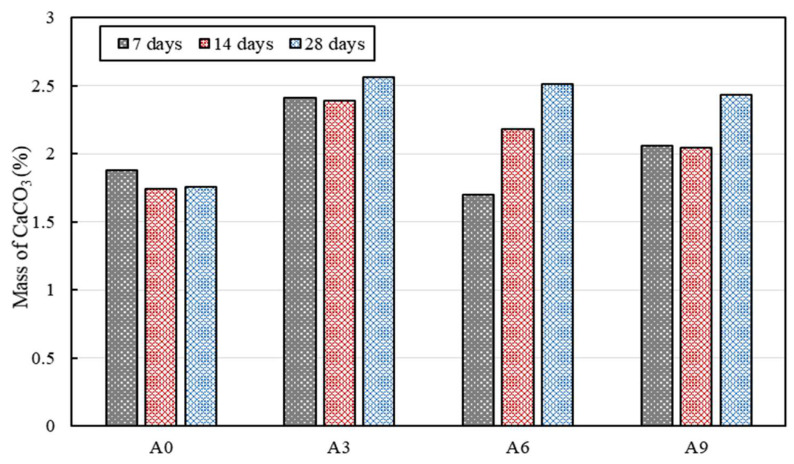
Mass of CaCO_3_ in the paste specimens on 7th, 14th, and 28th days, quantified from TG results.

Figure 6 and Figure 7 show the amount of portlandite and CaCO_3_ produced by TG analysis. Since portlandite and CaCO_3_ decomposed between 440 °C and 500 °C and between 680 °C and 760 °C, respectively, the amount of CaCO_3_ and portlandite was measured quantitatively by computing the weight loss in these two regions from the TG curves [35]. The incorporation of the AE agent to the culture medium significantly affected the amount of CaCO_3_ as well as portlandite. The highest amount of portlandite was formed when 0.3% AE agent was added. The increased formation of portlandite shows that the hydration was influenced by the addition of the AE agent, considering that calcium hydroxide is closely related to the hydration of C_3_S and C_2_S, and hence formation of C–S–H gels. On the 14th and 28th days, the highest amount of portlandite formation was observed when 0.3% AE agent was added, and the amount of portlandite increased compared with that on the 7th day.

The amount of CaCO_3_ increased when the AE agent was added on the 7th, 14th, and 28th days. It is reasonable to assume that the AE agent has a positive effect on the survivability of *S. pasteurii*, considering that the amount of CaCO_3_ produced increased as the number of viable *S. pasteurii* increased (Figure 2). The evidence suggests that bacteria can survive with a high pH of concrete and that CaCO_3_ can be precipitated, as in the previous study [23]. Although the survivability of *S. pasteurii* increased with increasing the AE agent content significantly, CaCO_3_ was highest in A3 containing 0.3% AE agent. This can be attributed to the chemical structure of the AE agent. The anionic surfactant AE agent used in this study has both hydrophilic and hydrophobic properties. In addition, it is involved in the interaction between air bubbles and particles by adsorbing to hydrates having various charges or adsorbing to negatively charged hydrates by utilizing the bridging effect of cationic metals [26]. In other words, the hydrophobic part of the AE agent surrounds the bacteria, and the other part, which is an anion, comes into contact with nutrients [31]. Therefore, it can be inferred that excessive incorporation of the AE agent inhibits CaCO_3_ formation since it prevents the access of *S. pasteurii* and nutrients [31]. Based on previous studies where bacteria survived in a urea–yeast medium at a pH of 12 or higher, and CaCO_3_ precipitated when sufficient calcium was supplied, it is thought that *S. pasteurii* that survived in the paste specimens may precipitate CaCO_3_ [31]. These results are consistent with previous studies which showed that the among calcium-based minerals (i.e., CaCO_3_, gypsum, ettringite, etc.), CaCO_3_ produced by bacterial metabolism affects the pore structure and reduces the total capillary porosity by acting as a filler [21,23].

### 3.4. Effect of Incorporation of an AE Agent on the Mechanical Properties of Mortar Specimens

**Figure 8:**
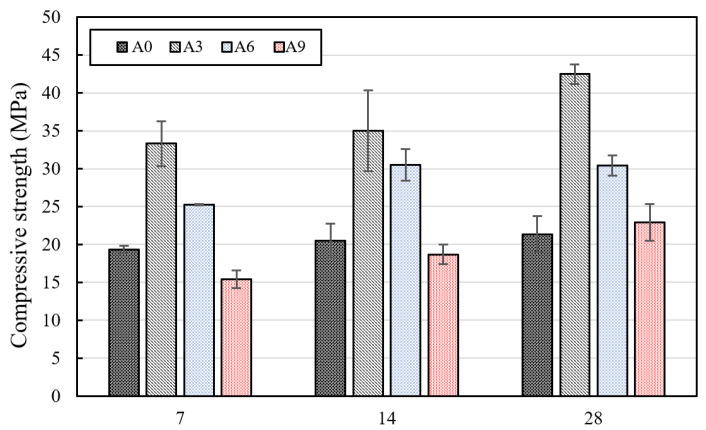
Compressive strengths of the mortar specimens on the 7th, 14th, and 28th days.

The compressive strengths of the mortar specimens are shown in Figure 8. The amount of incorporated AE agent significantly influenced the compressive strength on the 7th, 14th, and 28th days. Although specimen A3, incorporating 0.3% AE agent, showed the highest compressive strength, the compressive strength decreased when 0.6 and 0.9% AE agent was incorporated. It should be noted that the compressive strength of mortar specimens with 0.9% AE agent decreased compared to the specimen without the AE agent. The highest compressive strength was obtained when 0.3% AE agent was incorporated at 7, 14, and 28 days, and the compressive strength tended to decrease when the mixing amount of the AE agent of 0.6 and 0.9% was increased. The increase in compressive strength was observed in the mortar specimens on the 14th and 28th days. It can be inferred that the main cause of the increase in compressive strength was the effect of the AE agent on the cementitious materials and the effect of induced calcium carbonate precipitation (MICP) on the pore structures.

It can be said that incorporating a small dosage of an AE agent can improve the dispersion of cement powder in the culture medium [36]. As a result, the hydration of the cement and the compressive strength can be improved [36]. It is noted that this tendency is the same as in a previous study in which the compressive strength and the fluidity increased when the optimal quantity of the AE agent was incorporated in OPC [36]. In addition, the decrease in the compressive strength of the specimens was similar to the results of previous studies where the compressive strength decreased when 0.6% or more AE agent was incorporated [36]. When 0.6% or more AE agent was mixed with mixing water, the amount of air entrained in the specimens increased excessively and thus the compressive strength decreased.

Second, it may be thought that the presence of *S. pasteurii* may have a positive effect on the compressive strength. This indicates that the dead cells played the same role as the fiber and the compressive strength improved [21,22]. Supporting results can be found in the study of Ghosh et al. [21,22] where the compressive strength of the concrete increased when a culture medium inoculated with *Shewanella* species was mixed into the concrete, since the bacterial cells acted as microfiber. In addition, it can be inferred that the reduction in the porosity of the specimens due to the CaCO_3_ produced by bacteria suggests that the compressive strength and durability improved [37,38]. In this study, the effects of incorporation of an AE agent on bacteria survival during the initial curing of specimens up to 14 days were investigated. Therefore, further studies are required to investigate the bacteria survival in specimens during long-term curing.

## 4. Conclusions

The present study investigated the effect of an AE agent on the bacterial viability and mechanical properties of cement paste and mortar specimens. Specimens were fabricated by incorporating 0, 0.3, 0.6, and 0.9% AE agent by the weight of cement. The results showed that the incorporation of the AE agent can contribute to improving the survivability of *S. pasteurii* as well as the compressive strength of the mortar and paste specimens containing biological admixture. The main findings of this study are summarized as follows:

1. No significant changes in the pH and zeta potential value were observed in the culture media incorporating the AE agent. It can be inferred that the incorporation of the AE agent into the culture media did not affect the growth and metabolism of the *S. pasteurii* significantly in that the value of the zeta potential and pH of the culture media were influenced by the OH^−^ formed from the urea degradation of *S. pasteurii*.
2. The highest compressive strength was achieved by the mortar specimen with a 0.3% AE agent. It can be said that incorporation of 0.3% of AE agent may accelerate the initial hydration by improving dispersion of cement particles. Nevertheless, the compressive strength of specimens with 0.6% and 0.9% AE agent was lower than that of specimen with 0.3% AE agent, due to the excessive entrained air in the mortar specimens.
3. As the amount of the AE agent increased, the viable number of *S. pasteurii* also increased in the paste specimens. It can be inferred that the air entrained by the AE agent has a positive effect on the viability of *S. pasteurii*. Since the AE agent can uniformly distribute air bubbles of a certain size in the cementitious materials, it may provide spaces in the cement matrix for bacterial spores to survive, increasing the bacterial CaCO_3_ precipitation property.

## Figures and Tables

**Table 1:** Chemical composition of OPC (ordinary Portland cement) used in this study.

(wt %)	CaO	SiO_2_	Al_2_O_3_	Fe_2_O_3_	SO_3_	LOI *
Cement	63.75	21.50	6.20	3.20	2.14	3.21

* Loss on ignition.

**Table 2:** Mix proportion of mortar specimens by mass ratio.

Specimen Code	Cement	Sand	Medium	Urea	Calcium Lactate	AE Agent	W/B ^a^
A0	1.00	1.00	0.4	0.008	0.008	0.000	0.4
A3	1.00	1.00	0.4	0.008	0.008	0.003	0.4
A6	1.00	1.00	0.4	0.008	0.008	0.006	0.4
A9	1.00	1.00	0.4	0.008	0.008	0.009	0.4

^a^ Water/binder ratio.

**Table 3:** Target gene and primer sequence for quantitative PCR.

Primer	Sequence(5′-3′)	Target Gene	Product Length (bp)	Amplification Efficiency	Y-Intercept	R^2^
PUre-F	TCG ACC GTA GCT TGA CAG TG	ureA, ureB, ureC genes	222	93.019	36.6619	0.999
PUre-R	GTC TTG TCG GGT TCG TTG AT					

## Data Availability

All the data is available within the manuscript.
